# Economic evaluations of predictive genetic testing: A scoping review

**DOI:** 10.1371/journal.pone.0276572

**Published:** 2023-08-02

**Authors:** Qin Xi, Shihan Jin, Stephen Morris

**Affiliations:** 1 Primary Care Unit, Department of Public Health and Primary Care, University of Cambridge, Cambridge, United Kingdom; 2 Department of Pharmaceutical and Health Economics, Leonard D. Schaeffer Center for Health Policy and Economics, School of Pharmacy, University of Southern California, Los Angeles, California, United States of America; University of Tampere, FINLAND

## Abstract

Predictive genetic testing can provide information about whether or not someone will develop or is likely to develop a specific condition at a later stage in life. Economic evaluation can assess the value of money for such testing. Studies on the economic evaluation of predictive genetic testing have been carried out in a variety of settings, and this research aims to conduct a scoping review of findings from these studies. We searched the PubMed, Web of Science, Embase, and Cochrane databases with combined search terms, from 2019 to 2022. Relevant studies from 2013 to 2019 in a previous systematic review were also included. The study followed the recommended stages for undertaking a scoping review. A total of 53 studies were included, including 33 studies from the previous review and 20 studies from the search of databases. A significant number of studies focused on the US, UK, and Australia (34%, 23%, and 11%). The most frequently included health conditions were cancer and cardiovascular diseases (68% and 19%). Over half of the studies compared predictive genetic testing with no genetic testing, and the majority of them concluded that at least some type of genetic testing was cost-effective compared to no testing (94%). Some studies stated that predictive genetic testing is becoming more cost-effective with the trend of lowering genetic testing costs. Studies on predictive genetic testing covered various health conditions, particularly cancer and cardiovascular diseases. Most studies indicated that predictive genetic testing is cost-effective compared to no testing.

## Background

Genetic testing is the analysis of human DNA, RNA, chromosomes, proteins and specific metabolites with the goal of detecting heritable disease-related genotypes, mutations, phenotypes or karyotypes for clinical purposes [1]. Genetic testing results for gene mutations can help bring more accurate and timely predictions or diagnoses of health conditions and produce better health outcomes [2]. Applications of genetic testing span different medical disciplines in various clinical contexts, among which predictive genetic testing is defined as ‘the use of a genetic test in an asymptomatic person to predict future risk of disease’ [3, 4]. Such testing allows pre-symptomatic identification of individuals at risk of a specific condition, especially for adult-onset and complex diseases, and will lead to reduced morbidity and mortality through early implementation of targeted screening, surveillance, and prevention [3, 4]. Predictive genetic screening and testing have long been used in a variety of disease prevention strategies [5, 6]. Given the quantity of genes with an established correlation with diseases increasing over the last 20 years, genetic testing technologies are now being offered for an increasing number of conditions, including rare inherited conditions, such as Pompe disease (PD), and multifactorial conditions such as hereditary breast and ovarian cancer (HBOC), Lynch Syndrome, and familial hypercholesterolaemia (FH) [7–10].

The economic evaluation of predictive genetic testing is critical as genetic testing has become a powerful tool in disease risk prediction and prevention, and is unlikely to be cost-neutral. Economic evaluation is defined as the comparative analysis of alternative courses of action in terms of both their costs and consequences [11, 12]. The cost-effectiveness of an intervention is assessed to ensure maximum health gain from limited available resources [12]. It combines clinical and epidemiological evidence for measuring health outcomes and economic evidence for analyzing costs. All economic evaluations assess outcomes and costs, but approaches to measuring and valuing the consequences of health interventions may differ [13, 14]. Health economic modeling is commonly utilized to inform healthcare decisions in various systems [15]. With the advancement of genetic technology, predictive genetic testing has the potential for future implementation on an unprecedentedly large scale [16]. Ensuring value for money could help inform decision-makers about the affordability and cost-effectiveness of predictive genetic testing strategies, thus informing its implementation.

Several systematic and scoping reviews for the economic evaluation of genetic screening, testing, and precision medicine have been carried out previously. Several systematic and scoping reviews of economic evaluations of genetic screening, testing, and precision medicine have been carried out previously. Early systematic reviews of economic evaluations of genetic testing were undertaken by Carlson et al. (2005) covering studies published during 1990–2004 and Djalalov et al. (2011) covering studies published during 2004–2009 [17, 18]. D’Andrea et al. (2015) conducted a systematic review of the literature on economic evaluations of genetic testing [19]. Johnson et al. (2022) conducted a systematic review of the methodological quality of economic evaluations in genetic screening and testing for monogenic disorders over the period 2013–2019 [11]. Kasztura et al. (2019) conducted a scoping review on the cost-effectiveness of precision medicine [20]. These studies evaluated previous studies from the methodological perspectives and focused less on study findings. They also did not disaggregate findings by health condition. The present study updates previous reviews, with the most recent search results. The aims of this study were to 1) demonstrate when, where and how economic evaluations of predictive genetic testing were carried out, and 2) summarize their conclusions by different health conditions. Further discussions to identify the gaps existing in the current research scope were also carried out.

## Methods

We followed Arksey and O’Malley’s framework for conducting scoping reviews in the following stages [21]. The study was registered on OSF Registries on August 3^rd^, 2022.

### Stage 1. Identifying the research question

This scoping review will review the current research findings. Specifically, we aim to cover the following research questions: 1) When, where, and how have economic evaluations of predictive genetic testing been carried out? 2) What are the conclusions drawn from these studies? Specifically, what are the health conditions of interest for predictive genetic testing? For each health condition of interest, what are the genes tested, the adverse event considered, and cost components included? We also identify gaps in knowledge in terms of the cost-effectiveness of predictive genetic testing.

### Stage 2. Identifying relevant studies

The literature search utilized the search terms from previous reviews [11]. We searched in PubMed, Web of Science, Embase, and Cochrane (See Supporting Information 1 in S1 File). We included studies published after Sep 10, 2019, which were not included in previous literature reviews [11]. The search was last supplemented on June 11, 2022. Duplications from different databases were deleted.

Studies had to meet the following criteria to be included in the review: 1) They were a complete economic evaluation, which is defined as ‘the comparative analysis of alternative courses of action in terms of both their costs and consequences [12]. 2) They focused on predictive genetic testing, which is defined as ‘the use of a genetic test in an asymptomatic person to predict future risk of disease’ [3]. 3) They were written in English. 4) They were published in peer-reviewed journals.

### Stage 3. Study selection

The first-round screening was based on titles and abstracts. Studies were excluded for one or more of the following reasons in the first-round screening: A. Not about genetic testing. B. Not about economics. C. Not human studies. D. Duplicates not automatically deleted.

The second-round screening was based on the full article from the selected articles during the first-round screening. Papers were excluded if: A. they were not complete economic evaluation [22] (a complete economic evaluation must include both costs and outcomes, and must include at least one alternative strategy as a comparator [14]). B. they included only diagnostic yield as the health outcome measurement [23]. C. they were pharmacogenetic testing studies [24], (defined as ‘studies of how the genome background is associated with drug resistance and how therapy strategy can be modified for a certain person to achieve benefit’ [25]. D. they only included testing for reproductive purposes [26], including preconception and prenatal testing. E. they only included viral or bacterial pathogens [27]. F. they were Literature reviews, protocols, commentaries or conference abstracts [28]. G. they included testing conducted only among the symptomatic population [29]. H. they included testing for somatic mutations [30]. I. they included polygenic risk scores [31]. J. they included economic evaluations on topics related to genetic testing, but not relevant to the aim of this study. E.g., the cost-effectiveness of different recruiting strategies of genetic testing [32]. K. the paper not written in English [33].

The selected articles from the search of databases, as well as identified pre-2019 papers in a previous literature review [11], both entered the final review process.

The first-round screening was carried out by the first reviewer (QX). The second-round screening was conducted by two reviewers (QX and SJ) independently. When disagreements occurred between the two reviewers, they met to explain and discuss reasons for inclusion or exclusion. All disagreements were resolved between the two reviewers and consensus was finally reached.

### Stage 4. Charting the data

The data extraction criteria were based on previous literature reviews on similar topics. The included studies were first characterized by country, year of publication, methods of evaluation, measurement of health outcomes, and modeling types. Other technical details, including perspective taken, time horizon, discount rate, willingness-to-pay (WTP) threshold, were also listed. After studies had been categorized by the health condition of interest, features including population tested, genes covered by testing, the intervention of interest, comparison, the adverse event considered, results, conclusions, and cost components, were analyzed. Extracted data was charted into two tables, respectively for key findings in all studies and methodological details in studies identified through the search of databases (See Supporting Information 2 and 3 in S1 File).

### Stage 5. Collating, summarizing and reporting the results

Results were presented in a narrative synthesis. Due to the heterogeneity of the included studies, considerable challenges would occur if the results from studies were combined quantitatively [34]. Thus, due to the limitation of time and resources, no further data analysis or meta regression was planned or undertaken. Studies were first summarized by characteristics, then categorized by health conditions of interest and their respective findings were analyzed.

## Results

### Overview of the search results

**Fig 1:**
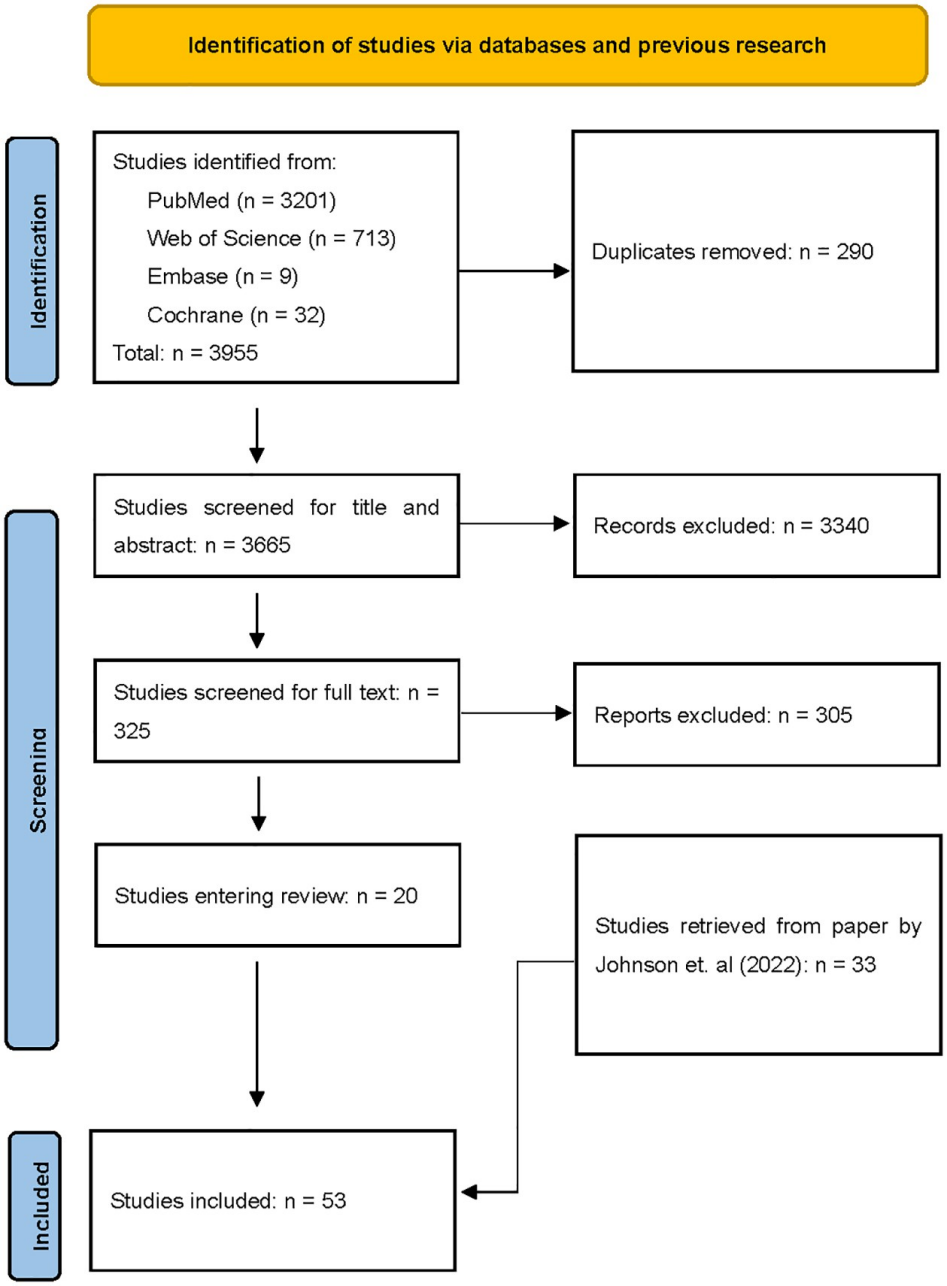
PRISMA diagram for study selection.

A total of 3955 papers were extracted from the database search, and 3665 studies were identified after automatically excluding the duplications. A total of 325 papers passed the first-round screening, which included titles and abstracts. After the second-rounding screening, which was based on the full article, 20 of the 325 papers were finally retained (Fig 1).

Studies included in the Johnson et al. paper (2022) were also screened based on the same inclusion and exclusion criteria. 33 papers featuring predictive genetic testing were included in further analysis [11]. Other studies were excluded since they focused on genetic testing for symptomatic populations, which could not be categorized as predictive testing.

53 studies entered the final review process (Fig 1). Details of selected studies are in Supporting Information 2 and 3 in S1 File.

### Characteristics of selected studies

**Fig 2:**
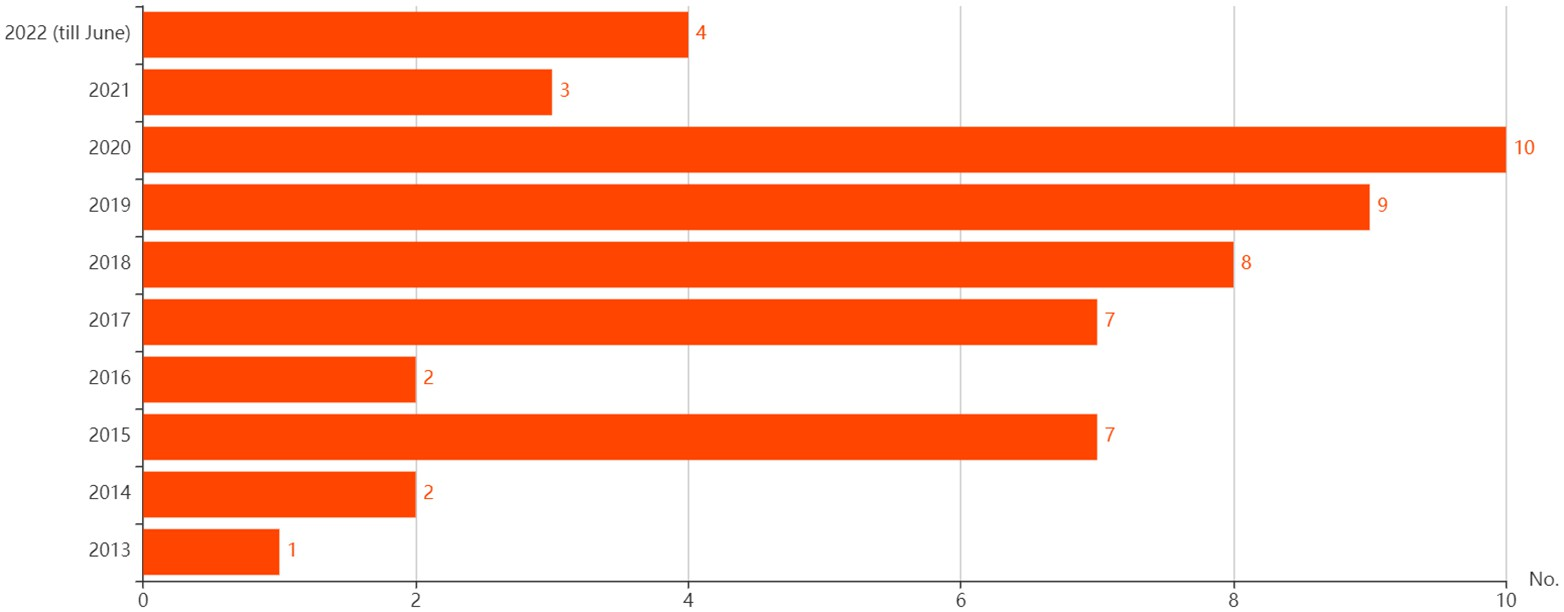
Number of publications per year.

Countries most frequently featured in the studies were the United States (n = 18), the United Kingdom (n = 12), and Australia (n = 6). Other countries included in the studies were Germany (n = 4), Netherlands (n = 3), Canada (n = 2), Brazil (n = 2), China (n = 2), Spain (n = 1), Norway (n = 1), Malaysia (n = 1), Israel (n = 1), India (n = 1), Taiwan (n = 1), Iran (n = 1), Poland (n = 1), Singapore (n = 1), Thailand (n = 1) and Switzerland (n = 1). Some studies included multiple-countries settings. There were publications in each year from 2013 to 2022. See Fig 2 for total publication numbers by year.

The methods for economic evaluation included cost-utility analysis (CUA), cost-effectiveness analysis (CEA), cost-consequence analysis (CCA), and cost-benefit analysis (CBA). Most studies were CUAs, or included a CUA alongside with other analysis methods (n = 41), the majority of which measured outcomes in terms of quality-adjusted life-years (QALYs) (n = 40); the other utilized disability-adjusted-life-year (DALY) (n = 1). Some papers included a CEA (n = 15), in which life-years (LYs) was utilized as the measurement for health outcomes. Other studies included a CCA (n = 2) and CBA (n = 1). Modeling methods used by the studies included decision trees (n = 33), Markov models (n = 27), and microsimulation modelling (n = 15). Some studies did not perform separate disease simulation, but rather drew data on health outcomes from published studies (n = 2). Summing the numbers in each category might not get the total number of studies since some studies included multiple methods. Other information, including length of follow-up period and perspectives taken by studies, are in Supporting Information 3 in S1 File.

### Health conditions covered by the studies

The majority of studies (n = 50) focused on testing for one type of germline genetic disorder and their related adverse events. Most health conditions covered by the studies were cancer-related (n = 36). The most frequently covered health condition of interest was BRCA1/BRCA2 genetic testing (n = 19) for hereditary breast and ovarian cancer (HBOC). Other topics featured in the studies include Lynch Syndrome (n = 13) for colorectal (CRC), endometrial (EC), and ovarian (OC) cancer; Li-Fraumeni Syndrome (n = 1) for adrenocortical carcinoma (ACC), etc.; Cowden Syndrome (n = 1) for thyroid cancer (TC), etc.; and pediatric cancer predisposition syndromes (n = 2) for medullary thyroid carcinoma (MTC), etc. Cardiovascular disease-related genetic mutations were also included in a variety of studies. (n = 10). The most common type of health condition of interest was familial hypercholesterolaemia (FH) (n = 8). Cardiomyopathy (n = 2) was also included, including both hypertrophic cardiomyopathy (HCM) and dilated cardiomyopathy (DCM). Other conditions of interest included Thrombophilia for venous thromboembolism (VTE) (n = 2), hereditary haemochromatosis (n = 1), and Pompe disease (PD) (n = 1).

The other studies covered multiple conditions (n = 3), featuring cancer-related, cardiovascular disease-related, and multiple other health conditions.

**Fig 3:**
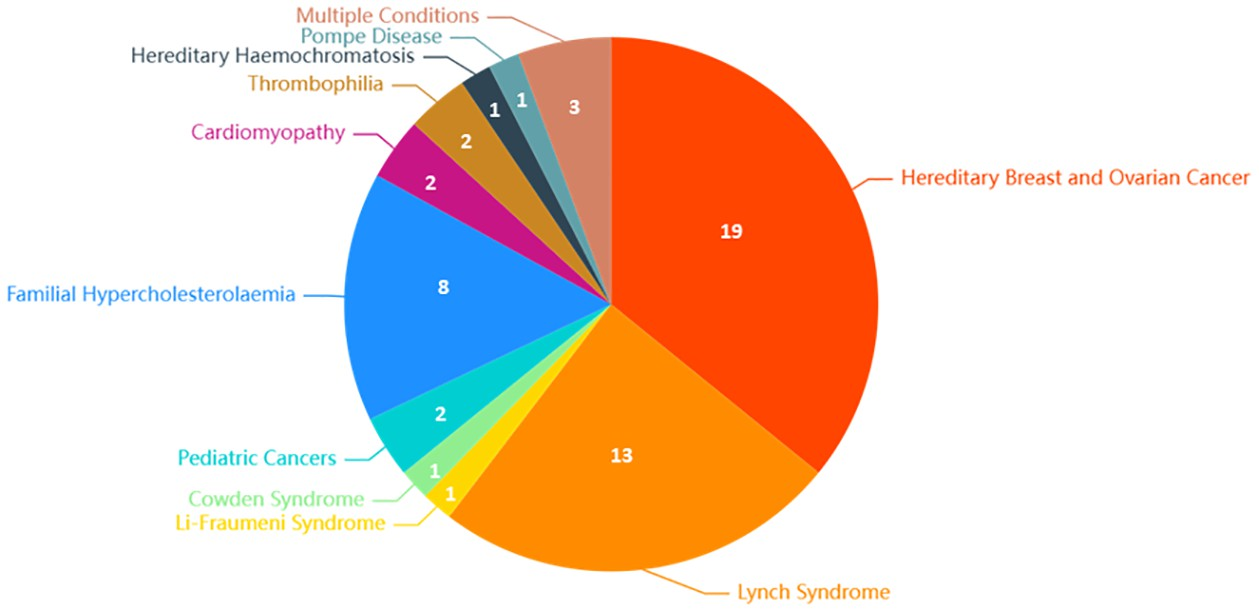
Number of publications by health condition.

See Fig 3 for a breakdown of publication numbers by health condition.

#### Hereditary Breast and Ovarian Cancer (HBOC) [35–53]

19 studies focused on genetic testing for the prevention of hereditary breast and ovarian cancer (HBOC). Studies featured monogenic screening for the risk prediction and prevention of HBOC. Most studies either featured patients diagnosed with breast and/or ovarian cancer and their female relatives if the patient tested positive or tested asymptomatic women with a family history of breast cancer (BC) and/or ovarian cancer (OC) (n = 13). A few studies conducted simulations on the entire unselected population, with or without a restriction for age (n = 4). The other studies tested for unselected women with (partly) Ashkenazi Jew ethnicity (n = 2). The majority of studies only utilized CUA (n = 15), all using QALYs as the measurement for health outcomes, while some others used both CUA and CEA (n = 3). One conducted a CCA (n = 1).

Three broad cost components were included in the studies: 1). Testing cost—costs incurred through genetic testing, including genetic counseling and genetic testing (for BRCA1/BRCA2/other genes mutation); 2) Prevention cost–costs incurred after genetic testing, but before the onset of disease, including the costs for risk reduction surgeries like risk-reducing salpingo-oophorectomy (RRSO), the costs for hormone replacement therapy (HRT), and the cost for BC and OC surveillance through regular screening; 3) Disease cost–costs incurred because of the onset of BC and OC, including cancer treatment, palliative or end of life care, as well as additional coronary heart disease (CHD) costs, and loss of productivity due to diseases in case of analysis from a societal perspective. Among the 19 studies, all studies included all three components (n = 19).

A number of studies compared some types of genetic testing with no genetic testing (n = 10), among which most studies used testing for BRCA1/BRCA2 as the testing strategy (n = 8), and the others tested for more genes (n = 2). Other studies compared the strategy of testing for multi-genes (7-/9-/14-gene panel) with only BRCA1/BRCA2 testing (n = 4). The remaining studies either compared population-based BRCA1/BRCA2 testing with family-history-based testing (n = 4), or made comparisons between different uptake rates of BRCA1/BRCA2 testing (n = 1). Featured genes included BRCA1, BRCA2, CDH1, PALB2, PTEN, STK11, TP53, ATM, BARD1, BRIP1, CHEK2, NBN, RAD51C, and RAD51D. All studies considered both BC and OC as adverse events in health outcomes.

Where this was the comparison, all studies concluded that some type of genetic testing was cost-effective compared to no genetic testing (n = 10). For studies multi-gene testing versus BRCA1/BRCA2 only testing, it was concluded that multi-gene testing was cost-effective (n = 4), although a 14-gene panel was considered not cost-effective compared to 7-gene panel testing. Some studies concluded that population-based testing was cost-saving compared to family history-based testing (n = 2). In contrast, other studies gave contradicting results (cost-saving, cost-effective, or not cost-effective) in different settings of country (n = 1) or age group (n = 1). Another study concluded the higher uptake rate of BRCA1/BRCA2 testing was cost-saving (n = 1).

#### Lynch Syndrome (LS) [54–66]

13 papers studied the predictive genetic testing of Lynch Syndrome (LS) for prediction and prevention of colorectal cancer (CRC), endometrial cancer (EC) and ovarian cancer (OC). The majority of studies tested patients diagnosed with CRC, and implemented cascade testing for their relatives if the patient tested positive (n = 10). One study tested for women diagnosed with EC, and tested for relatives if the patients is positive (n = 1). The other studies tested for an unselected population (n = 2). More than half of the studies were CEAs (n = 7), while the others were either CUAs (n = 5), or both CUA and CEA (n = 1).

Among all studies, the costs included could be categorized into 3 parts: 1) Lynch Syndrome diagnostics, including implementation of various risk assessment criteria (Amsterdam, Bethesda, etc.), immunohistochemistry (IHC) testing for MMR protein expression and BRAP, MLH1 hypermethylation analysis, as well as genetic testing and counseling; 2) Cancer surveillance after diagnosis of Lynch Syndrome, including colonoscopy, and gynaecologic surveillance and prophylactic surgery in relatives, as well as chemoprevention with Aspirin; 3) CRC and/or EC costs, including cancer treatment and palliative therapy. All included the diagnostics and surveillance costs, including cancer treatment and palliative therapy (n = 13), including cancer treatment and palliative therapy.

The majority of studies compared genetic testing with no testing or screening (n = 9). A few studies compared different age groups or different selection standards when it comes to the target testing population (n = 3). Another study made comparisons between Next-Generation Sequencing (NGS) and the current standard of care, as well as different ranges of genes tested in NGS (n = 1). The testing methods covered in the comparisons included immunohistochemistry (IHC) testing, microsatellite instability (MSI) testing, which is used to detect possible DNA mismatch repair (MMR) defect, and testing for BRAF mutation and MLH1 methylation, as well as NGS. All studies covered CRC as an adverse event (n = 13), some studies also covered EC (n = 6), while only a few studies considered the health and economic outcomes caused by OC (n = 2).

Though the conclusions drawn by the studies differ by types of comparison and population tested, most studies tended to conclude that some type of genetic testing was cost-effective compared to no genetic testing (n = 8). One study found that LS screening provided clinical benefit but at a high cost (n = 1). Also, NGS was considered to be cost-effective compared to the current standard of care (n = 1). Studies also concluded that expanding the age group for genetic testing, so that older age groups are also tested was cost-effective (n = 2). Another study concluded that population screening might be cost-effective in younger patient populations with a relatively inexpensive test cost compared to family history-based testing (n = 1).

#### Li-Fraumeni Syndrome [67]

See Supporting Information 4 in S1 File

#### Cowden Syndrome [68]

See Supporting Information 4 in S1 File

#### Pediatric Cancers [69, 70]

See Supporting Information 4 in S1 File

#### Familial Hypercholesterolaemia (FH) [71–78]

Eight studies covered the predictive genetic testing for familial hypercholesterolemia (FH). Most studies tested the relatives of FH patients (n = 5), some studies covered unselected children aged 1–2 years old (n = 2), and the other study tested for multiple populations, including first job takers, people who have experienced an acute coronary syndrome (ACS) event (and relatives if positive), and children aged 6 years old (n = 1). The adverse event considered include coronary artery disease (CAD), stroke, myocardial infarction (MI), stable angina, unstable angina, and heart failure. Most studies included only a CUA (n = 6), while 2 included both a CUA and a CEA (n = 2).

Cost components included 1) Screening, including genetic and lipid testing; 2) Follow-up and treatment (medication cost for lipid-lowering statin treatment and statin adherence program, etc.); and 3) Costs for cardiovascular disease (CVD) events. Most studies clearly described their cost components (n = 7). One study additionally included indirect costs for analysis from societal perspective.

The majority of studies compared genetic testing with no testing (n = 7), while another study compared genetic testing with lipid screening (n = 1). Most studies provided details on the adverse events (n = 7). All studies found that at least one of the listed genetic testing strategies for FH was cost-effective compared to no genetic testing, across various healthcare systems in different countries and among various population groups (n = 7). One study concluded that genetic testing was not cost-effective compared to lipid screening (n = 1).

#### Cardiomyopathy [79, 80]

See Supporting Information 4 in S1 File

#### Thrombophilia [81, 82]

See Supporting Information 4 in S1 File

#### Hereditary Haemochromatosis (HH) [83]

See Supporting Information 4 in S1 File

#### Pompe Disease (PD) [84]

See Supporting Information 4 in S1 File

#### Multiple Conditions [85–87]

See Supporting Information 4 in S1 File

## Discussion

### 1. Summary of main findings

In our review, predictive genetic testing was used in research for various health conditions and from different countries and health systems. The research identified made comparisons between different genetic testing strategies. Different target populations were selected, including general populations in a certain age group or those with a family history of a certain condition. Different genes were included for similar health conditions, like the BRCA1/BRCA2 testing and 14-gene panel testing for HBOC. The economic evaluation method also varied, including CUA, CEA, CCA, and CBA. Modeling techniques included in the studies were decision tree, Markov modeling, and microsimulation. A cohort study was also used. Cost components included in each study vary widely, but most could still fit in the general classification of testing, prevention, and disease costs. Studies from different geographic areas used different WTP thresholds with different currencies.

Among the 35 studies that make comparisons between genetic testing and no genetic testing, 33 of them concluded that at least one strategy including genetic testing was cost-effective. Though types of comparisons differ by health conditions, populations, and interventions, such findings provide evidence of the health economic value of implementing predictive genetic testing.

### 2. Implications

Due to the disparities in research scope, a definite and comprehensive conclusion about the cost-effectiveness of predictive genetic strategies was hard to achieve, even within the range of the same health condition of interest. The choice of comparators could affect the conclusions on whether genetic testing is cost-effective. The comparators in each study were different, but can be generalized into two categories: 1) no genetic testing, and 2) another type of genetic testing. Though it would be hard to quantify how the comparators affect the conclusions, it could be concluded that if the genetic testing strategy is compared to no genetic testing, the intervention would be considered more relatively expensive and effective, while if the genetic testing strategy is comparison to another type of genetic testing, the intervention would be considered less relatively expensive and effective.

However, most studies did conclude with a positive attitude towards the role of genetic testing in disease risk prevention and indicated that genetic testing is cost-effective compared to no genetic testing, though the optimal testing strategy would vary depending on various factors, including the disease type, costs of testing, etc. Some studies that resulted in less positive attitudes toward genetic testing also indicated that the cost-effectiveness of genetic testing could be increased with the lowering cost of genetic technologies.

### 3. Compliance with clinical guidelines

We noticed that the studies did not indicate that they follow clinical guidelines strictly for comparators and interventions. Due to the complex nature of clinical practices, it’s reasonable to simplify scenarios for the simplicity of modelling. Still, it’s worth discussing that to which extent should economic evaluations follow the clinical guidelines of the specific country or region in future studies.

As indicated in Supporting Information 2 in S1 File, some studies follow the clinical guideline by using guideline-based current practice (e.g., testing cancer patients and their first-degree relatives) as the comparator. Other studies do not strictly follow the clinical guideline by not using standard care as the comparator. When it comes to the follow-up interventions on individuals, in most studies, in both the genetic testing and no genetic testing scenario, the interventions applied were simplified compared to clinical guidelines. For example, for Hereditary breast and ovarian cancer (HBOC) in the UK settings, some studies assume an option of Risk-Reducing Mastectomy (RRM) and Risk-Reducing Salpingo-Oophorectomy (RRSO) for those tested positive for genetic mutation. By comparison, according to the NICE clinical guideline, there are detailed prevention and surveillance implementation options according to the individual’s age, family history, and a number of other factors [88]. Those details are not implicated in the cost-effectiveness studies.”

### 4. Strengths and limitations

The study was the first to comprehensively summarize current findings drawn from previous studies on the economic evaluation of predictive genetic testing. It provided the most updated information on the current research landscape, across different health conditions, health care systems, and methodologies for evaluation.

There are several limitations. First, due to the limited resources and time, the first-round screening for titles and abstracts was only conducted by one author. A comprehensive double screening method could bring more reassuring results. Another limitation of the study was that a previous review was used to identify pre-2019 studies. Also, the study did not examine and compare the methodological details of each study, including the time horizon, discount rate, etc., nor examined the comprehensiveness and quality of each research. Such details might have influenced the outcomes of each study.

When it comes to the importance of the results, we would suggest caution in generalizing conclusions from different studies. First, there is still lack of data in certain fields, which affects the accuracy of certain studies. For example, few studies have considered the effect of false positive results in genetic testing, and the psychological impact it would cause. Second, though we analyzed the cost components for each study, detailed items within each cost component were often not the same across studies, even for the same health conditions. For example, some studies included end-of-life care costs in disease costs [49], while others did not [35]. This could lead to difference in the calculation of costs, and might affect conclusions when they are close to the threshold. Third, different studies modelled disease status differently. For example, some studies modelled different stages of cancer and used varies parameters for different stages [48], some studies modelled germline and sporadic cancers as different statuses [52], while others simply modelled the disease as one status and use the same set of parameters for all patients. Though these modelling methods should ideally lead to similar results, the possibility that they affect the comparability of conclusions is not neglectable. Fourth, the willingness-to-pay (WTP) threshold is not the same in each study within each country. Different WTP threshold could lead to different conclusions when it comes to whether the interventions are cost-effective. Fifth, the intervention strategies are different between studies, even for similar health conditions. This is obvious in studies concerning Lynch syndrome, in which various strategies are used as interventions and comparators. Sixth, demographic difference of genetic information between ethnicities could make conclusions from certain studies not reliable in another country with different demographic distribution. Current studies are mostly from countries where Caucasian people form the majority of population, and relies heavily on the genetic data of people with Caucasian ethnicity. Such conclusions are not representable for the global population. Those factors led to considerable heterogeneity between studies, and formed considerable obstacles for researchers to advance the analysis with a meta-analysis.

Several other factors have also affected people from giving generalized advice to policy on the implementation of genetic testing based on included studies. First and foremost, the application of genetic information in the prevention of different diseases are in different stages. There have been well-established genetic information-based prevention strategies in hereditary breast and ovarian cancer (HBOC), but not other health conditions. This might be due to various reasons, for example, the lack of intervention options available in the case of Alzheimer’s disease. Health gains will be different if there is or isn’t a cure (prevention strategy) for the disease. Second, there is considerable geographic disparity. While some countries make health policy decisions based on health economic evaluations (the UK and Canada), others view such evaluations more as academic exploration rather than evidence for policy making (the US).

### 5. Further research

The study gives light on future studies on economic evaluations of predictive genetic testing. There are a variety of other diseases (Huntington’s disease, Alzheimer’s disease, etc.), or other health outcomes related to a genetic mutation (Lynch Syndrome-related prostate cancer), for which genetic testing is clinically utilized for risk prediction, but for which there is little or no economic evidence. Also, the combination of genetic testing with more recent technologies, like polygenic risk scores (PRS), could provide more information on the cost-effectiveness of genetic technologies and guide utilization [89]. Moreover, this review indicates a clear disparity in the geographic distribution of related studies, with Europe and North America making up most of the studies. Other regions are under-represented in this field of study. With the emergence of more data and research resources, studies on other diseases, combining other technologies and with settings in different countries, could contribute to the comprehensiveness of the research landscape on the economic evaluations of predictive genetic testing.

## Conclusions

Studies on predictive genetic testing covered a wide variety of health conditions, with particular focus on cancer and cardiovascular diseases. Though the heterogeneity of the studies presents considerable obstacle to providing generalized conclusions, we could see certain consensus drawn from studies that used similar comparators–for example, the studies that compare genetic testing to no genetic testing demonstrated the economic value of genetic testing technologies. Among studies that compare predictive genetic testing with no genetic testing, the majority indicated that genetic testing is cost-effective, providing evidence for the economic feasibility of predictive genetic testing. Other studies stated that with the lowering cost of genetic technology, predictive genetic testing is becoming a more generally cost-effective technology. Future research could focus on the comparisons between or the combination of predictive genetic testing and other genetic technologies for disease risk prediction, including polygenic risk score (PRS).

## Supporting information

S1 Checklist PRISMA 2020 checklist. (DOCX)

S1 File (DOCX)
