# Supplementary material for: Economic evaluations of predictive genetic testing: A scoping review
Source: PLoS One. 2023 Aug 2;18(8):e0276572. doi: 10.1371/journal.pone.0276572 (PMC10395838; doi:10.1371/journal.pone.0276572)
Supplement: S1 File — (DOCX) [file pone.0276572.s002.docx]

**Supporting Information**

*The Supporting Information uses a different References list from the manuscript. The numbering system for citations is separate.*

Supporting Information 1. Search terms

The databases and search terms used in this study was consistent with a previous research, with updated time range (1).

**PubMed:**

((("Genetic Testing"[Mesh] AND "Cost-Benefit Analysis"[Mesh]) OR ("Genetic Testing/economics"[Mesh]) OR ("Genetic Carrier Screening"[Mesh] AND "Cost-Benefit Analysis"[Mesh])) OR (("mutation test" OR "mutation tests" OR "mutation testing" OR "mutation testings" OR "mutation screen" OR "mutation screening" OR "mutation screens" OR "mutation screenings" OR "genetic test" OR "genetic testing" OR "genetic tests" OR "genetic testings" OR genetic screen* OR "personalized medicine" OR population test* OR population screen* OR genomic diagnostic test* OR "next-generation sequencing" OR "whole-exome sequencing" OR "whole exome sequencing" OR "whole genome sequencing") AND ("cost effective" OR "cost-effective" OR "cost-effectiveness analysis" OR "cost effectiveness" OR "cost-effectiveness" OR "economic evaluation" OR "cost-utility" OR "cost utility" OR "cost-benefit" OR cost benefit*))) AND ( "2019/09/10"[PDat] : "3000/12/31"[PDat])

SPECIES: Humans & LANGUAGE: English & TEXT AVAILABILITY: Full text

**Web of Science:**

TOPIC: ("mutation test*" OR "mutation screen*" OR "genetic test*" OR "genetic screen*" OR "carrier screen*" OR "personalized medicine" OR "population test*" OR "population screen*" OR "genomic diagnostic test*" OR "next-generation sequencing" OR "whole-exome sequencing" OR "whole exome sequencing" OR "whole-genome sequencing" OR "whole genome sequencing") AND ("cost-effective*" OR "cost effective*" OR "economic evaluation" OR "cost-utility" OR "cost utility" OR "cost-benefit" OR "cost benefit")

Document Type: Article & Language: English & Publication Date: 2019-09-10 to 3000-12-31

**Embase:**

('mutation test/exp' OR 'mutation screen/exp' OR 'genetic test'/exp OR 'genetic screen'/exp OR 'carrier screen'/exp OR 'personalized medicine' OR 'population test'/exp OR 'population screen'/exp OR 'genomic diagnostic test'/exp OR 'next-generation sequencing' OR 'whole-exome sequencing' OR 'whole exome sequencing' OR 'whole-genome sequencing' OR 'whole genome sequencing' OR 'genetic screening'/de OR 'next-generation sequencing'/de OR 'whole exome sequencing'/de) AND ('cost-effective'/exp OR 'cost effective'/exp OR 'economic evaluation'/exp OR 'cost-utility' OR 'cost utility' OR 'cost-benefit' OR 'cost benefit' OR 'cost-effectiveness analysis'/de OR 'cost utility'/de OR 'cost benefit'/de) NOT [medline]/lim

Years: 2019 to 2022

**Cochrane:**

([mh "genetic testing"] OR [mh "genetic carrier screening"] OR [mh "whole genome sequencing"] OR “mutation test*” OR “mutation screen*” OR "genetic test*" OR “genetic screen*” OR “carrier screen*” OR "personalized medicine" OR "population test*" OR "population screen*" OR "genomic diagnostic test*" OR "next-generation sequencing" OR "whole-exome sequencing" OR "whole exome sequencing" OR "whole genome sequencing" OR “whole-genome sequencing”) AND ([mh "cost-benefit analysis"] OR "cost-effective*" OR "cost effective*" OR "economic evaluation" OR cost-utility OR "cost utility" OR cost-benefit OR "cost benefit")

Date: 10/09/2019 to 31/12/2300 & In Trials

Supporting Information 2. Contents of included studies

| **Health Condition** | **Author** | **Year** | **Country** | **Population** | **Intervention** | **Comparison** | **Measurement** | **Method** | **WTP threshold** | **Result** | **Conclusion** |
| --- | --- | --- | --- | --- | --- | --- | --- | --- | --- | --- | --- |
| Hereditary Breast and Ovarian Cancer (HBOC) | Asphaug et al. (2) | 2019 | Norway | patients with breast cancer <60 (and 1st degree female relatives if positive) | 7-gene panel | BRCA 1/2 testing | QALY | CUA | USD 77,000 | USD 53,310 | 7-gene panel is cost-effective, compared to BRCA 1/2 testing |
|  |  |  |  |  | 14-gene panel | 7-gene panel |  |  |  | USD 127,071 | 14-gene panel is not cost-effective, compared to 7-gene panel testing |
|  | Eccleston et al. (3) | 2017 | UK | patients with epithelial ovarian cancer (and ﬁrst- and second-degree relatives if positive) | germline BRCA 1/2 testing | no genetic testing | QALY | CUA | GBP 20,000 | GBP 4,339 | germline BRCA 1/2 testing is cost-effective |
|  | Hoskins et al. (4) | 2019 | Canada | patients with epithelial ovarian cancer (and ﬁrst- and second-degree relatives if positive) | BRCA 1/2 testing | no genetic testing | QALY | CUA | CAD 100,000 | CAD -8,919 | germline BRCA 1/2 testing is cost-saving |
|  | Kemp et al. (5) | 2019 | UK | cancer patients meeting Mainstreaming Cancer Genetics (MCG) criteria | 9-gene panel | no genetic testing | QALY | CUA | USD 26,184 | USD 1,330 | 9-gene panel is cost-effective |
|  |  |  |  | cancer patients meeting MCGplus criteria |  |  |  |  |  | USD 1,225 |  |
|  | Li et al. (6) | 2017 | US | 40-year-old asymptomatic women with family history of breast or ovarian cancer or other hereditary syndromes such as Li-Fraumeni syndrome and Cowden syndrome that predispose to breast cancer | 7-gene panel | BRCA 1/2 testing | QALY | CUA |  | USD 48,328 | 7-gene panel followed by risk-reduction management is cost-effective |
|  |  |  |  |  |  |  | LY | CEA | USD 100,000 | USD 23,734 |  |
|  |  |  |  | 50-year-old asymptomatic women with family history of breast or ovarian cancer or other hereditary syndromes such as Li-Fraumeni syndrome and Cowden syndrome that predispose to breast cancer | 7-gene panel | BRCA 1/2 testing | QALY | CUA |  | USD 69,920 |  |
|  |  |  |  |  |  |  | LY | CEA | USD 100,000 | USD 42,067 |  |
|  | Lim et al. (7) | 2018 | Malaysia | patients with breast cancer | BRCA 1/2 testing | no genetic testing | QALY | CUA | USD 9,500 | USD 2,725 | BRCA 1/2 testing is cost-effective |
|  | Manchanda et al. (8) | 2018 | UK | all women | FH-based BRCA1/BRCA2/RAD51C/RAD51D/BRIP1/PALB2 testing | clinical criteria/FH-based BRCA 1/2 testing | QALY | CUA | GBP 30,000 | GBP 7629.25 | population-based testing for BRCA1/BRCA2/RAD51C/RAD51D/BRIP1/PALB2 mutations is the most cost-effective |
|  |  |  |  |  | BRCA1/BRCA2/RAD51C/RAD51D/BRIP1/PALB2 testing | clinical criteria/FH-based BRCA1/BRCA2/RAD51C/RAD51D/BRIP1/PALB2 testing |  |  |  | GBP 21,599.96 |  |
|  |  |  | US |  | FH-based BRCA1/BRCA2/RAD51C/RAD51D/BRIP1/PALB2 testing | clinical criteria/FH-based BRCA 1/2 testing |  |  | USD 100,000 | USD 49,282.19 |  |
|  |  |  |  |  | BRCA1/BRCA2/RAD51C/RAD51D/BRIP1/PALB2 testing | clinical criteria/FH-based BRCA1/BRCA2/RAD51C/RAD51D/BRIP1/PALB2 testing |  |  |  | USD 54,769.78 |  |
|  | Manchanda et al. (9) | 2015 | UK | Ashkenazi women >30 | BRCA 1/2 testing | FH-based testing | QALY | CUA | GBP 20,000 | GBP -2,079 | population screening is cost-saving |
|  | Manchanda et al. (10) | 2017 | UK | women with 1-4 Ashkenazi Jewish grandparent(s) | BRCA 1/2 testing | FH-based testing | QALY | CUA | GBP 20,000 | GBP (-2,960) - 863 | population testing for BRCA mutations with varying levels of Ashkenazi-Jewish ancestry is cost-effective in the United Kingdom and the United States. |
|  |  |  | US |  |  |  |  |  | USD 100,000 | USD (-19,587) - (-2,542) |  |
|  | Muller et al. (11) | 2018 | Germany | women aged 35 with BRCA 1/2 mutation probability >= 10% | BRCA 1/2 testing | no genetic testing | QALY | CUA | multiple | EUR 17,027 | genetic testing to high-risk women with a BRCA1 and two mutation probability of ≥ 10% based on the individual family cancer history appears to be a cost-effective option |
|  |  |  |  |  |  |  | LY | CEA |  | EUR 22,318 |  |
|  | Tuffaha et al. (12) | 2018 | Australia | female patients aged 40 with breast cancer with >10% risk of BRCA variants (and ﬁrst- and second-degree relatives if positive) | BRCA 1/2 testing | no genetic testing | QALY | CUA | AUD 50,000 | AUD 18,900 | BRCA testing in women with breast cancer is cost-effective. extending testing to cover family members of affected women who test positive improves cost-effectiveness |
|  |  |  |  |  | cascade testing | no genetic testing |  |  |  | AUD 14,900 |  |
|  | Neusser et al. (13) | 2019 | Germany | female relatives of hereditary breast cancer patients | 90% uptake in BRCA 1/2 testing | 9% uptake in BRCA 1/2 testing |  | CCA |  |  | cost savings due to breast and ovarian cancer treatment in the scenario of rising demands |
|  | Michaan et al. (14) | 2020 | Israel | all women | BRCA 1/2 testing | no genetic testing | QALY | CUA | NIS 340,000 | NIS 81,493 (2.5% prevalence rate) | BRCA 1/2 testing is cost-effective |
|  | Guzauskas et al. (15) | 2020 | US | 30-year-old women | population BRCA 1/2 testing | FH-based testing | QALY | CUA | USD 100,000 | USD 87,700 | population HBOC screening may be cost-effective among younger women but not among older women |
|  |  |  |  | 30-year-old women (and 1st degree female relatives if positive) |  |  |  |  |  | USD 92,600 |  |
|  |  |  |  | 45-year-old women |  |  |  |  |  | USD 268,200 |  |
|  |  |  |  | 45-year-old women (and 1st degree female relatives if positive) |  |  |  |  |  | USD 354,500 |  |
|  | Hurry et al. (16) | 2019 | Canada | patients with epithelial ovarian cancer and breast cancer (first-/second-degree relatives tested if index patient/first-degree relative is positive) | BRCA 1/2 testing | no genetic testing | QALY | CUA | CAD 100,000 | CAD 14,942 | prevention via testing and RRS is cost effective |
|  | Correa-Galendi et al. (17) | 2020 | Brazil | 30-year-old first- and second-degree relatives of women with BRCA-related cancer | BRCA 1/2 testing | no genetic testing | QALY | CUA | R 25,000 | BRL 24,263 | BRCA 1/2 testing for women with high risk is cost-effective |
|  |  |  |  |  |  |  | LY | CEA |  | BRL 27,258 |  |
|  | Sun et al. (18) | 2022 | China | patients with breast cancer (and ﬁrst- and second-degree relatives if positive) | BRCA1/BRCA2/PALB2 testing | no genetic testing | QALY | CUA | USD 10,260 | USD 4,506 | unselected multigene testing is cost-effective |
|  |  |  |  |  |  |  |  |  |  | USD 7,266 |  |
|  | Sun et al. (19) | 2019 | UK | patients with breast cancer (and ﬁrst- and second-degree relatives if positive) | BRCA1/BRCA2/PALB2 testing | FH-based BRCA 1/2 testing | QALY | CUA | GBP 30,000 | GBP 7,216 | unselected multigene testing is cost-effective |
|  |  |  |  |  |  |  |  |  |  | GBP 10,464 |  |
|  |  |  | US |  |  |  |  |  | USD 100,000 | USD 61,618 |  |
|  |  |  |  |  |  |  |  |  |  | USD 65,661 |  |
|  | Manchanda et al. (20) | 2020 | UK | all women >= 30 | BRCA 1/2 testing | FH-based testing | QALY | CUA | USD 42,656/28,471 | USD -5,639 | population-based testing is cost-saving |
|  |  |  | US |  |  |  |  |  | USD 57,589/50,000 | USD -4,018 | population-based testing is cost-saving |
|  |  |  | Netherlands |  |  |  |  |  | USD 50,539/24,390 | USD -11,433 | population-based testing is cost-saving |
|  |  |  | China |  |  |  |  |  | USD 15,531 | USD 18,066 | population-based testing is cost-effective |
|  |  |  | Brazil |  |  |  |  |  | USD 15,182 | USD 13,579 | population-based testing is cost-effective |
|  |  |  | India |  |  |  |  |  | USD 6,574 | USD 23,031 | population-based testing is not cost-effective |
|  |  |  | UK |  |  |  |  |  | USD 42,656/28,471 | USD 21,191 | population-based testing is highly cost-effective |
|  |  |  | US |  |  |  |  |  | USD 57,589/50,000 | USD 16,552 | population-based testing is highly cost-effective |
|  |  |  | Netherlands |  |  |  |  |  | USD 50,539/24,390 | USD 25,215 | population-based testing is highly cost-effective |
|  |  |  | China |  |  |  |  |  | USD 15,531 | USD 23,485 | population-based testing is cost-effective |
|  |  |  | Brazil |  |  |  |  |  | USD 15,182 | USD 20,995 | population-based testing is cost-effective |
|  |  |  | India |  |  |  |  |  | USD 6,574 | USD 32,217 | population-based testing is not cost-effective |
| Lynch Syndrome | Barzi et al. (21) | 2015 | US | CRC patients, general population | predictive model + IHC + germline testing | no screening | LY | CEA | USD 50,000 | USD 35,143 | the initial step in screening for Lynch Syndrome should be the use of predictive models in probands. universal tumor testing and general population screening strategies are not cost-effective |
|  |  |  |  |  | IHC + BRAF + germline testing |  |  |  |  | USD 144,117 |  |
|  |  |  |  |  | universal germline testing of colon cancer probands |  |  |  |  | USD 996,878 |  |
|  | Chen et al. (22) | 2016 | Taiwan | patients newly diagnosed with CRC and first-degree relatives of LS probands | IHC + BRAF (if MLH1 is absent) + gene sequencing (if BRAF mutation not found) | no screening | LY | CEA | USD 50,000 | USD 6,025 | the strategy based on immunohistochemistry as a genetic test followed by BRAF mutation testing was considered to be highly cost-effective relative to no screening |
|  |  |  |  |  | IHC + gene sequencing |  |  |  |  | USD 7,088 |  |
|  |  |  |  |  | MSI + gene sequencing |  |  |  |  | USD 23,872 |  |
|  |  |  |  |  | gene sequencing |  |  |  |  | USD 145,110 |  |
|  | Gallego et al. (23) | 2015 | US | patients referred to a cancer genetics clinic and relatives if positive | NGS panel testing (with Lynch syndrome genes and other genes associated with highly penetrant CRCP syndromes) | current standard of care | QALY | CUA | USD 100,000 | USD 36,500 | the use of an NGS panel that includes genes associated with highly penetrant CRCP syndromes in addition to Lynch syndrome genes as a ﬁrst-line test is likely to provide meaningful clinical beneﬁts in a cost-effective manner |
|  |  |  |  |  | NGS panel testing (with Lynch syndrome genes, other genes associated with highly penetrant CRCP syndromes, and genes with low colorectal cancer penetrance) | NGS panel testing (with Lynch syndrome genes and other genes associated with highly penetrant CRCP syndromes) |  |  |  | USD 77,300 |  |
|  | Gansen et al. (24) | 2019 | Germany | CRC patients and relatives of LS mutation carriers | counseling including Bethesda + IHC + BRAF + sequencing | no screening | LY | CEA |  | EUR 46,494 | higher LS test uptakes could have effects on the lives and rights of colorectal cancer patients and their relatives. privacy limitations could yield health gains for ﬁrst-degree relatives of index patients and substantially improve cost-effectiveness but this approach may contradict the right to informational self-determination. |
|  |  |  |  |  | counseling + IHC + BRAF + sequencing |  |  |  |  | EUR 154,431 |  |
|  |  |  |  |  | counseling + direct sequencing |  |  |  |  | EUR 2,213,028 |  |
|  | Goverde et al. (25) | 2017 | Netherlands | patients <= 70 years old with EC (and relatives if positive) | screening in EC patients <= 70 (MSI, IHC, and germline DNA analysis) | screening in EC patients <50 | LY | CEA | EUR 40,000 | EUR 5,252 | routine LS screening in EC patients ≤70 years is a cost-effective strategy, allowing colorectal cancer prevention in EC patients and their relatives |
|  |  |  |  |  |  | screening based on revised Bethesda guidelines |  |  |  | EUR 6,668 |  |
|  | Leenen et al. (26) | 2016 | Netherlands | patients <= 70 years old with CRC (and relatives if positive) | screening CRC patients ≤60 years | screening CRC patients ≤50 years | LY | CEA | EUR 40,000 | EUR 4,226 | routine LS screening by analysis of microsatellite instability, immunohistochemistry, and MLH1 hypermethylation in CRC patients ≤70 years of age is a cost-effective strategy with important clinical benefits for CRC patients and their relatives. |
|  |  |  |  |  | screening CRC patients ≤70 years | screening CRC patients ≤60 years |  |  |  | EUR 7,051 |  |
|  |  |  |  |  |  | screening based on revised Bethesda guidelines |  |  |  | EUR 7,341 |  |
|  | Severin et al. (27) | 2015 | Germany | patients with newly-diagnosed CRC and their ﬁrst-degree relatives | Bethesda criteria + IHC + BRAF + genetic sequencing | no screening | LY | CEA | EUR 50,000 | EUR 77,268 | LS screening provides clinical benefit but at a high cost. the most cost-effective strategy involves family-history assessment with the revised Bethesda criteria, followed by IHC testing, BRAF testing, and genetic sequencing. chemoprevention appears to provide comparably low additional benefit and improves cost-effectiveness only slightly |
|  |  |  |  |  | IHC + BRAF + genetic sequencing | Bethesda criteria + IHC + BRAF + genetic sequencing |  |  |  | EUR 253,258 |  |
|  |  |  |  |  | direct sequencing of all index patients | IHC + BRAF + genetic sequencing |  |  |  | EUR 4,188,036 |  |
|  | Snowsill et al. (28) | 2015 | UK | individuals (aged <50) with newly-diagnosed early-onset CRC (not metachronous CRC) and their relatives | MSI + BRAF (if MSI found) + genetic testing (if BRAF mutation not found) | no testing | QALY | CUA | GBP 20,000 | GBP 5,491 | reflex testing for Lynch syndrome in early-onset colorectal cancer patients is predicted to be a cost-effective use of limited financial resources in England and Wales |
|  |  |  |  |  | IHC + genetic testing (if IHC abnormal) / MSI + BRAF + genetic testing (if IHC normal) | MSI + BRAF (if MSI found) + genetic testing (if BRAF mutation not found) |  |  |  | GBP 25,106 |  |
|  |  |  |  |  | direct genetic testing | IHC + genetic testing (if IHC abnormal) / MSI + BRAF + genetic testing (if IHC normal) |  |  |  | GBP 82,962 |  |
|  | Pastorino et al. (29) | 2020 | Italy | individuals with newly-diagnosed colorectal cancer and ﬁrst-degree relatives if positive | Bethesda criteria + IHC + MS-MLPA analysis (for patients >=70) | no screening | QALY | CUA | EUR 30,000 | EUR 941.24 | universal testing versus no testing is cost-effective, but not necessarily in comparison with age-targeted strategies |
|  |  |  |  |  | IHC + MS-MLPA analysis |  |  |  |  | EUR 1,026.83 |  |
|  |  |  |  |  | genetic sequencing |  |  |  |  | EUR 1,681.93 |  |
|  | Kang et al. (30) | 2020 | Australia | people with incident CRC and at-risk relatives | IHC + BRAF | no testing | LY | CEA | AUD 30,000-50,000 | AUD 28,915 | universal tumor testing strategies for guiding germline genetic testing of people with incident CRC for LS in Australia are likely to be cost-effective compared with no testing. universal germline gene panel testing would not currently be cost- effective. |
|  |  |  |  |  | IHC |  |  |  |  | AUD 227,000 |  |
|  |  |  |  |  | universal gene panel testing |  |  |  |  | AUD 2,411,933 |  |
|  | Guzauskas et al. (31) | 2022 | US | unselected population | screening unselected 30-year-olds | FH-based screening | QALY | CUA | USD 150,000 | USD 132,151 | population LS screening may be cost-effective in younger patient populations under a $150,000 willingness-to-pay per QALY threshold and with a relatively inexpensive test cost |
|  |  |  |  |  | screening unselected 50-year-olds | FH-based screening |  |  |  | USD 140,371 |  |
|  | Snowsill et al. (32) | 2020 | UK | women with EC and relatives of LS patients | MSI | no testing | QALY | CUA | GBP 20,000-30,000 | Dominant | universal testing for Lynch syndrome in endometrial cancer patients is expected to be cost-eﬀective in the U.K. (NHS), and the Manchester approach is expected to be the optimal testing strategy |
|  |  |  |  |  | MSI + MLH1 methylation | MSI |  |  |  | GBP 3,738 |  |
|  |  |  |  |  | IHC + MLH1 methylation | MSI + MLH1 methylation |  |  |  | GBP 5,459 |  |
|  |  |  |  |  | direct NGS | IHC + MLH1 methylation |  |  |  | Dominant |  |
|  | Azardoost et al. (33) | 2021 | Iran | patients with CRC | Amsterdam + IHC + MSI + NGS | no testing | QALY | CUA |  | USD 5,337 | IHC testing followed by NGS testing could be regarded as the most cost-effective strategy compared to the other strategies |
|  |  |  |  |  |  |  | LY | CEA |  | USD 6,029 |  |
|  |  |  |  |  | Amsterdam + IHC + NGS |  | QALY | CUA |  | USD 4,604 |  |
|  |  |  |  |  |  |  | LY | CEA |  | USD 5,202 |  |
|  |  |  |  |  | Amsterdam + MSI + IHC + NGS |  | QALY | CUA |  | USD 5,763 |  |
|  |  |  |  |  |  |  | LY | CEA |  | USD 6,511 |  |
|  |  |  |  |  | Amsterdam + MSI + NGS |  | QALY | CUA |  | USD 4,748 |  |
|  |  |  |  |  |  |  | LY | CEA |  | USD 5,364 |  |
|  |  |  |  |  | Amsterdam + NGS |  | QALY | CUA |  | USD 10,639 |  |
|  |  |  |  |  |  |  | LY | CEA |  | USD 12,043 |  |
| Li-Fraumeni Syndrome | Kunst et al. (34) | 2022 | US | newborns | population-wide screening for TP53 variants | usual care | LY | CEA | USD 100,000 | USD 106,009 | TP53-NBS could be cost-effective |
| Cowden Syndrome | Ngeow et al. (35) | 2015 | US | CS-like patients | PTEN CC score as a clinical risk calculator to identity for PTEN germline testing | no use of PTEN germline testing | QALY | CUA | USD 100,000 | USD 58,884-107,390 (male) and USD 50,569-155,367 (female) | use of the CC score as a clinical risk calculator is a cost-effective prescreening method to identify CS-like patients for PTEN germline testing |
| Pediatric Cancers | Yeh et al. (36) | 2021 | US | newborns | genetic testing for pediatric cancer predisposition syndromes (CPS) | no testing | QALY | CUA | USD 100,000 | USD 99,430 | population-based genetic testing of newborns may reduce mortality associated with pediatric cancers and could be cost-effective as sequencing costs decline |
|  | O'Brien et al. (37) | 2022 | US | siblings of newborns with cancer susceptibility gene variants (RET, RB1, TP53, DICER1, SUFU, PTCH1, SMARCB1, WT1, APC, ALK, or PHOX2B) | cascade testing | usual care | LY | CEA |  | USD 16,910 | sibling cascade testing would enhance newborn screening efforts and targeted screening approaches may be more efficient than universal screening to achieve population-level benefits. |
| Familial Hypercholesterolaemia (FH) | Ademi et al. (38) | 2014 | Australia | first- and second-degree relatives of FH patients | cascade screening | no screening | QALY | CUA | AUD 6,000 | AUD 3,565 | cascade screening for FH, using genetic testing supplemented with the measurement of plasma low-density lipoprotein cholesterol concentrations and treatment with statins, is a cost-effective means of preventing CHD in families at risk of FH |
|  |  |  |  |  |  |  | LY | CEA |  | AUD 4,155 |  |
|  | Chen et al. (39) | 2014 | US | Caucasian male adults with family history of FH | genetic cascade screening | lipid cascade screening | QALY | CUA | USD 150,000 | USD 519,813 | genetic screening is currently not a cost-effective option in the US, health outcomes for FH individuals could beneﬁt from adherence measures encouraging statin use. |
|  |  |  |  |  | lipid cascade screening with statin adherence program | lipid cascade screening |  |  |  | USD 12,223 |  |
|  | Crosland et al. (40) | 2018 | UK | potential FH cases and relatives | clinical assessment with DLCN criteria | no genetic testing | QALY | CUA | GBP 20,000 | GBP 3,254 | searching primary care databases for people at high risk of FH followed by cascade testing is likely to be cost-effective. combined possible and definite SB criteria is slightly more cost effective than the standard DLCN criteria. |
|  |  |  |  |  | clinical assessment with SB criteria | clinical assessment with Dutch Lipid Clinic Network (DLCN) criteria |  |  |  | GBP 13,365 |  |
|  |  |  |  |  | secondary care identification with DLCN criteria | no genetic testing |  |  |  | Dominant |  |
|  |  |  |  |  | secondary care identification with BS criteria |  |  |  |  | Dominant |  |
|  |  |  |  |  | secondary & primary care identification with DLCN criteria |  |  |  |  | GBP 82,338 |  |
|  |  |  |  |  | secondary & primary care identification with BS criteria |  |  |  |  | GBP 63,514 |  |
|  | Kerr et al. (41) | 2017 | UK | relatives of FH patients | cascade testing | no cascade testing | QALY | CUA | GBP 20,000 - 30,000 | GBP 5,806 | cascade testing of relatives of those with suspected FH is highly cost effective. |
|  | Lazaro et al. (42) | 2017 | Spain | FH patients and relatives | FH testing and cascade screening (the national program for FH - NPFH) | usual clinical care | QALY | CUA | EUR 30,000 | EUR 29,608 | The NPFH based on molecular testing is a cost-effective diagnostic and management strategy |
|  |  |  |  |  |  |  |  |  |  | Dominant |  |
|  | McKay et al. (43) | 2018 | UK | 1-2 year olds | cholesterol-only screening | no screening | QALY | CUA | GBP 20,000 | GBP 19,298 | a strategy involving cholesterol screening followed by diagnostic genetic testing and RCT is the most cost-effective modelled. implementation of universal cholesterol screening followed by diagnostic genetic testing and RCT for FH, under a UK conventional willingness-to-pay threshold is supported. |
|  |  |  |  |  | sequential cholesterol-genetic screening |  |  |  |  | GBP 21,872 |  |
|  |  |  |  |  | sequential cholesterol-genetic screening plus RCT |  |  |  |  | GBP 12,480 |  |
|  |  |  |  |  | sequential genetic-cholesterol screening |  |  |  |  | GBP 283,799 |  |
|  |  |  |  |  | sequential genetic-cholesterol screening plus RCT |  |  |  |  | GBP 84,240 |  |
|  |  |  |  |  | parallel cholesterol-genetic screening |  |  |  |  | GBP 131,635 |  |
|  |  |  |  |  | parallel cholesterol-genetic screening plus RCT |  |  |  |  | GBP 63,957 |  |
|  | Pelczarska et al. (44) | 2018 | Poland | those after an acute coronary syndrome (ACS) event (with genetic test) | cascade screening if positive | no screening | QALY | CUA | EUR 29,800 | EUR 817 | screening ACS patients below 55/65 years of age in men/women is the most cost-effective strategy. reducing age limits or using genetic tests reduce cost-effectiveness. all strategies are cost-effective |
|  |  |  |  |  |  |  | LY | CEA |  | EUR 1,049 |  |
|  |  |  |  | those after an acute coronary syndrome (ACS) event before 55 in men and 65 in women (with genetic test) |  |  | QALY | CUA |  | EUR 4,329 |  |
|  |  |  |  |  |  |  | LY | CEA |  | EUR 4,727 |  |
|  |  |  |  | first job takers (with genetic test) |  |  | QALY | CUA |  | EUR 1,528 |  |
|  |  |  |  |  |  |  | LY | CEA |  | EUR 1,650 |  |
|  |  |  |  | children |  |  | QALY | CUA |  | EUR 1,371 |  |
|  |  |  |  |  |  |  | LY | CEA |  | EUR 1,476 |  |
|  | Martin et al. (45) | 2021 | Australia | children 1-2 years of age (parent and first-degree relative if positive) | total cholesterol (TC) test and genetic testing if positive | no screening | QALY | CUA | AUD 28,033 | AUD 3,979 | universal screening of children aged 1–2 years for FH, undertaken at the time of an immunization, was a feasible and effective approach to detect children, parents and other blood relatives with FH |
| Cardiomyopathy | Catchpool et al. (46) | 2019 | Australia | 18-year-old asymptomatic relatives of patients with DCM | cascade genetic testing prior to periodical clinical surveillance | periodical clinical surveillance | QALY | CUA | AUD 40,000-70,000 | AUD 6,100 | using cascade genetic testing to guide clinical surveillance of asymptomatic relatives of patients with DCM is very likely to be cost-effective |
|  | Brough et al. (47) | 2020 | US | military population | echocardiogram only | no screening |  | CCA |  |  | the main barriers for the implementation of genetic screening for the U.S. military are the low detection rate and variant interpretation |
|  |  |  |  |  | genetic testing only |  |  |  |  |  |  |
|  |  |  |  |  | genetic screening; positive genetic test followed by echocardiogram |  |  |  |  |  |  |
| Thrombophilia | Compagni et al. (48) | 2013 | Italy | women with a familial history of VTE seeking oral contraception | genetic screening for the predisposition to Venous Thromboembolism | no genetic screening | QALY | CUA | EUR 40,000-50,000 | EUR 72,412 | testing strategy is cost-ineffective and leads to an overall loss of QALY |
|  | Sutherland et al. (49) | 2019 | Switzerland | first- time seekers of a CHC | Pill Protect (PP) genetic screening test | standard of care | QALY | CUA | CHF 100,000 | CHF 76,610 | PP test may be cost effective in Switzerland for screening women seeking CHCs for their risk of VTE based on the current evidence |
| Hereditary Haemochromatosis (HH) | Graaf et al. (50) | 2017 | Australia | males (30 years) and females (45 years) of northern European ancestry | screening for HFE C282Y variant homozygosity | cascade or incidental screening | QALY | CUA | AUD 50,000 | AUD 15,233 (males) 10,195 (females) | genotyping and TfS strategies are likely to be more cost-effective screening strategies than the status quo |
| Pompe Disease | Richardson et al. (51) | 2020 | US | newborns | universal Screening | no screening | QALY | CUA | USD 150,000-450,000 | USD 408,000 | newborn screening for Pompe disease results in substantial health gains for individuals with infantile-onset Pompe disease, but with additional costs |
|  |  |  |  |  |  |  |  |  |  | USD 379,000 |  |
| Multiple Conditions | Bennette et al. (52) | 2015 | US | patients with cardiomyopathy | returning incidental findings from next-generation genomic sequencing | not returning findings | QALY | CUA | USD 100,000 | USD 44,800 | returning incidental findings is likely cost-effective for certain patient populations. screening for generally healthy individual is not likely cost-effective. |
|  |  |  |  | patients with CRC |  |  |  |  |  | USD 115,020 |  |
|  |  |  |  | healthy individuals |  |  |  |  |  | USD 58,600 |  |
|  | Zhang et al. (53) | 2018 | US | young adults | genomic screening | no screening | DALY | CUA | AUD 50,000 | AUD 4,038 | preventive genomic screening in early adulthood would be highly cost-effective in a single-payer health-care system |
|  | Jittikoon et al. (54) | 2020 | Thailand | / | genetic testing | no genetic testing |  | CBA |  |  | genetic testing gained much more benefits than the cost |

Supporting Information 3. Methodologies of studies from the search of databases

| Author | Model Type | Time Horizon | Discount Rate | Perspective |
| --- | --- | --- | --- | --- |
| Pastorino et al. (29) | Decision Tree, Markov Model | Lifetime | 3.5% | Healthcare System |
| Jittikoon et al. (54) | / | Lifetime | 3.0% | Provider, Societal |
| Brough et al. (47) | / | / | / | / |
| Michaan et al. (14) | Decision Tree, Markov Model | Lifetime | 3.0% | Healthcare System |
| Guzauskas et al. (15) | Decision Tree, Markov Model | Lifetime | 3.0% | Healthcare System |
| Kang et al. (30) | Microsimulation | Lifetime | 5.0% | Provider |
| Yeh et al. (36) | Microsimulation | Lifetime | 3.0% | Societal |
| Hurry et al. (16) | Microsimulation | 50 Years | 1.5% | Healthcare System |
| Sutherland et al. (49) | Microsimulation | Lifetime | 5.0% | Healthcare System, Payer, Societal |
| O'Brien et al. (37) | Microsimulation | Lifetime | 3.0% | Societal |
| Guzauskas et al. (31) | Decision Tree, Markov Model | Lifetime | 3.0% | Societal |
| Kunst et al. (34) | Microsimulation | 15 Years | 3.0% | Societal |
| Martin et al. (45) | / | / | / | Healthcare System |
| Correa-Galendi et al. (17) | Decision Tree, Markov Model | Lifetime | 5.0% | Healthcare System |
| Richardson et al. (51) | Microsimulation | Lifetime | 3.0% | Societal, Healthcare System |
| Sun et al. (18) | Microsimulation | Lifetime | 3.0% | Payer, Societal |
| Sun et al. (19) | Microsimulation | Lifetime | 3.5% | Payer, Societal |
| Manchanda et al. (20) | Markov Model | Lifetime | 3.0% | Payer, Societal |
| Snowsill et al. (32) | Decision Tree, Markov Model | Lifetime | 3.5% | Healthcare System |
| Azardoost et al. (33) | Decision Tree, Markov Model | Lifetime | 3.0% | Healthcare System |

Supporting Information 4. Health conditions with ≤3 publications

***Li-Fraumeni Syndrome (34)***

One study covered the genetic testing for Li-Fraumeni Syndrome (LFS) for the prevention of adrenocortical carcinoma (ACC), choroid plexus carcinoma (CPC), osteosarcoma (OS), and rhabdomyosarcoma (RMS). The cost components included genetic testing, cancer surveillance, and cancer treatment. The study carried out an economic evaluation of newborn screening for TP53 mutation among unselected populations, compared to no genetic testing. It concluded that the TP53 newborn screening was cost-effective with a USD 100,000 WTP threshold.

***Cowden Syndrome (35)***

One study covered the genetic testing for Cowden Syndrome (CS) with thyroid cancer (TC), renal cancer (kidney cancer, KC), endometrial cancer (EC), and breast cancer (BC) as possibly related adverse events. It evaluated the cost-utility of carrying out a PTEN germline testing among CS-like patients compared to no germline testing. Cost components included consultation, test, intervention, and productivity loss. The cost-effectiveness ratio was USD 58,884-107,390 per QALY gained for male and USD 50,569-155,367 per QALY gained for female, making it possibly cost-effective under USD 100,000 WTP threshold.

***Pediatric Cancers (36, 37)***

Two studies covered the genetic testing for pediatric cancer predisposition syndromes. One study featured unselected population compared with no testing (n = 1). The cost components were genetic testing, cancer surveillance, and cancer treatment. Another study evaluated siblings of newborns with cancer susceptibility gene variants compared with usual care (n = 1). The cost component included consultation, test, intervention, and productivity loss.

Both studies tested for the same genes: RET, RB1, TP53, DICER1, SUFU, PTCH1, SMARCB1, WT1, APC, ALK, or PHOX2B (n = 2), for the prevention of a variety of pediatric cancers, including medullary thyroid carcinoma (MTC), retinoblastoma, adrenocortical carcinoma (ACC), choroid plexus, rhabdomyosarcoma (RMS), osteosarcoma, rhabdoid tumors (RT), pleuropulmonary blastoma (PPB), medulloblastoma, neuroblastoma, Wilms’ tumor, hepatoblastoma. Compared with usual care, both studies conclude that genetic testing for pediatric cancer was cost-effective (n = 2).

***Cardiomyopathy (46, 47)***

Two studies included testing for cardiomyopathy. One study tested unaffected 18-year-old, first-degree relatives of patients with dilated cardiomyopathy (DCM) (n = 1), and compared periodical clinical surveillance with and without cascade genetic testing. It included costs of exome sequencing and clinical surveillance (consultation with a cardiologist, electrocardiography, and echocardiography). It utilized a CUA and concluded that using cascade genetic testing to guide clinical surveillance of asymptomatic relatives of patients with DCM was very likely to be cost-effective.

Another study focused on the US military population for hypertrophic cardiomyopathy (HCM) (n = 1). It compared the combination of genetic testing and echocardiogram with no testing and included costs of genetic testing and echocardiogram, and training costs of personnel, etc. It was a CCA, and the main conclusion was that the main barriers to the implementation of genetic screening for the U.S. military were the low detection rate and variant interpretation.

***Thrombophilia (48, 49)***

Two studies tested for the genetic mutations for thrombophilia leading to Venous Thromboembolism (VTE) among asymptomatic female individuals. One study focused on women with a risk/family history of VTE (n = 1), and another study tested for unselected women seeking hormone contraception (n = 1). Cost components included 1) Testing costs (n = 2), and 2) Adverse event costs (n = 2), including hospitalization and pharmacological costs caused by pulmonary embolism (PE), deep vein thrombosis (DVT), and severe bleeding. Both studies used CUA for economic evaluation (n = 2).

Studies reached different conclusions regarding the cost-effectiveness of genetic testing for thrombophilia compared with no genetic testing (n = 1), or standard care (n = 1). One study concluded that it may be cost-effective compared to standard of care (n =1), while another study concluded that genetic testing is not cost-effective compared to no genetic screening and leads to an overall loss of QALY (n = 1).

***Hereditary Haemochromatosis (HH) (50)***

One study focused on the genetic testing for hereditary haemochromatosis (HH). It evaluated screening for HFE C282Y variant homozygosity, compared with cascade or incidental screening, for males aged 30 years males and females aged 45 years of northern European ancestry. Costs included screening tests, medical consultations, procedures, investigations, prescribed medications, and public hospital admissions. CUA was utilized as the evaluation method, and the testing was considered to be cost-effective.

***Pompe Disease (PD) (51)***

One study focused on the genetic testing for Pompe disease. Universal newborn screening was evaluated, in contrast with no screening. Cost components are surveillance and treatment, genetic testing and counseling, and condition costs. The evaluation method was CUA. The conclusion was that newborn screening for Pompe disease resulted in substantial health gains for individuals with infantile-onset Pompe disease, but with additional costs. The strategy might be cost-effective under the relatively loose USD 150,000-450,000 WTP threshold for rare diseases.

***Multiple Conditions (52-54)***

One study tested jointly for various conditions, including HBOC, Lynch Syndrome, FH, HCM, DCM, arrhythmogenic right ventricular cardiomyopathy (ARVC), malignant hyperthermia susceptibility (MHS), and Romano-Ward long QT syndrome (LQTS) types 1, 2, and 3. The population tested included patients with cardiomyopathy, patients with CRC, and healthy individuals, respectively, in separate simulations. Returning incidental findings was compared with not returning findings, and costs included surveillance and treatment, genetic testing and counseling, and condition costs. CUA was utilized. It concluded that returning incidental findings was likely to be cost-effective for specific patient populations, but screening for the generally healthy individual was not expected to be cost-effective.

One study tested for multiple cancers (HBOC, Lynch Syndrome-related CRC and EC), and various preconception carrier screenings. Costs included cancer screening, preconception carrier screening, combined screening for cancers or other conditions. CUA was adopted as the method for economic evaluation. It concluded that preventive genomic screening in early adulthood would be highly cost-effective in a single-payer healthcare system.

One study tested for HBOC, MI and Cardiomyopathy, and used a top-down approach for cost calculation. CBA was used for economic evaluation. The results showed that genetic testing gained much more benefits than the cost, and concluded that genetic testing was cost-beneficial, compared to no genetic testing.

References:

1. Johnson K, Saylor K, Guynn I, Hicklin K, Berg JS, Lich KH. A systematic review of the methodological quality of economic evaluations in genetic screening and testing for monogenic disorders. Genet Med. 2022;24(4):969.

2. Asphaug L, Melberg HO. The Cost-Effectiveness of Multigene Panel Testing for Hereditary Breast and Ovarian Cancer in Norway. MDM Policy Pract. 2019;4(1):2381468318821103.

3. Eccleston A, Bentley A, Dyer M, Strydom A, Vereecken W, George A, et al. A Cost-Effectiveness Evaluation of Germline BRCA1 and BRCA2 Testing in UK Women with Ovarian Cancer. Value Health. 2017;20(4):567-76.

4. Hoskins P, Eccleston A, Hurry M, Dyer M. Targeted surgical prevention of epithelial ovarian cancer is cost effective and saves money in BRCA mutation carrying family members of women with epithelial ovarian cancer. A Canadian model. Gynecol Oncol. 2019;153(1):87-91.

5. Kemp Z, Turnbull A, Yost S, Seal S, Mahamdallie S, Poyastro-Pearson E, et al. Evaluation of Cancer-Based Criteria for Use in Mainstream BRCA1 and BRCA2 Genetic Testing in Patients With Breast Cancer. JAMA Netw Open. 2019;2(5):e194428.

6. Li Y, Devlin JJ. A multigene test could cost-effectively help extend life expectancy for women at risk of hereditary breast cancer-Reply to letter to the editor by Petelin et al. Value Health. 2018;21(7):893-4.

7. Lim KK, Yoon SY, Mohd Taib NA, Shabaruddin FH, Dahlui M, Woo YL, et al. Is BRCA Mutation Testing Cost Effective for Early Stage Breast Cancer Patients Compared to Routine Clinical Surveillance? The Case of an Upper Middle-Income Country in Asia. Appl Health Econ Health Policy. 2018;16(3):395-406.

8. Manchanda R, Patel S, Gordeev VS, Antoniou AC, Smith S, Lee A, et al. Cost-effectiveness of Population-Based BRCA1, BRCA2, RAD51C, RAD51D, BRIP1, PALB2 Mutation Testing in Unselected General Population Women. J Natl Cancer Inst. 2018;110(7):714-25.

9. Manchanda R, Legood R, Burnell M, McGuire A, Raikou M, Loggenberg K, et al. Cost-effectiveness of population screening for BRCA mutations in Ashkenazi jewish women compared with family history-based testing. J Natl Cancer Inst. 2015;107(1):380.

10. Manchanda R, Patel S, Antoniou AC, Levy-Lahad E, Turnbull C, Evans DG, et al. Cost-effectiveness of population based BRCA testing with varying Ashkenazi Jewish ancestry. Am J Obstet Gynecol. 2017;217(5):578 e1- e12.

11. Muller D, Danner M, Schmutzler R, Engel C, Wassermann K, Stollenwerk B, et al. Economic modeling of risk-adapted screen-and-treat strategies in women at high risk for breast or ovarian cancer. Eur J Health Econ. 2019;20(5):739-50.

12. Tuffaha HW, Mitchell A, Ward RL, Connelly L, Butler JRG, Norris S, et al. Cost-effectiveness analysis of germ-line BRCA testing in women with breast cancer and cascade testing in family members of mutation carriers. Genet Med. 2018;20(9):985-94.

13. Neusser S, Lux B, Barth C, Pahmeier K, Rhiem K, Schmutzler R, et al. The budgetary impact of genetic testing for hereditary breast cancer for the statutory health insurance. Curr Med Res Opin. 2019;35(12):2103-10.

14. Michaan N, Leshno M, Safra T, Sonnenblick A, Laskov I, Grisaru D. Cost Effectiveness of Whole Population BRCA Genetic Screening for Cancer Prevention in Israel. Cancer Prev Res (Phila). 2021;14(4):455-62.

15. Guzauskas GF, Garbett S, Zhou Z, Spencer SJ, Smith HS, Hao J, et al. Cost-effectiveness of Population-Wide Genomic Screening for Hereditary Breast and Ovarian Cancer in the United States. JAMA Netw Open. 2020;3(10):e2022874.

16. Hurry M, Eccleston A, Dyer M, Hoskins P. Canadian cost-effectiveness model of BRCA-driven surgical prevention of breast/ovarian cancers compared to treatment if cancer develops. Int J Technol Assess Health Care. 2020;36(2):104-12.

17. Simoes Correa-Galendi J, Del Pilar Estevez Diz M, Stock S, Muller D. Economic Modelling of Screen-and-Treat Strategies for Brazilian Women at Risk of Hereditary Breast and Ovarian Cancer. Appl Health Econ Health Policy. 2021;19(1):97-109.

18. Sun L, Cui B, Wei X, Sadique Z, Yang L, Manchanda R, et al. Cost-Effectiveness of Genetic Testing for All Women Diagnosed with Breast Cancer in China. Cancers (Basel). 2022;14(7).

19. Sun L, Brentnall A, Patel S, Buist DSM, Bowles EJA, Evans DGR, et al. A Cost-effectiveness Analysis of Multigene Testing for All Patients With Breast Cancer. JAMA Oncol. 2019.

20. Manchanda R, Sun L, Patel S, Evans O, Wilschut J, De Freitas Lopes AC, et al. Economic Evaluation of Population-Based BRCA1/BRCA2 Mutation Testing across Multiple Countries and Health Systems. Cancers (Basel). 2020;12(7).

21. Barzi A, Sadeghi S, Kattan MW, Meropol NJ. Comparative effectiveness of screening strategies for Lynch syndrome. J Natl Cancer Inst. 2015;107(4).

22. Chen YE, Kao SS, Chung RH. Cost-Effectiveness Analysis of Different Genetic Testing Strategies for Lynch Syndrome in Taiwan. PLoS One. 2016;11(8):e0160599.

23. Gallego CJ, Shirts BH, Bennette CS, Guzauskas G, Amendola LM, Horike-Pyne M, et al. Next-Generation Sequencing Panels for the Diagnosis of Colorectal Cancer and Polyposis Syndromes: A Cost-Effectiveness Analysis. J Clin Oncol. 2015;33(18):2084-91.

24. Gansen F, Severin F, Schleidgen S, Marckmann G, Rogowski W. Lethal privacy: Quantifying life years lost if the right to informational self-determination guides genetic screening for Lynch syndrome. Health Policy. 2019;123(10):1004-10.

25. Goverde A, Spaander MC, van Doorn HC, Dubbink HJ, van den Ouweland AM, Tops CM, et al. Cost-effectiveness of routine screening for Lynch syndrome in endometrial cancer patients up to 70years of age. Gynecol Oncol. 2016;143(3):453-9.

26. Leenen CH, Goverde A, de Bekker-Grob EW, Wagner A, van Lier MG, Spaander MC, et al. Cost-effectiveness of routine screening for Lynch syndrome in colorectal cancer patients up to 70 years of age. Genet Med. 2016;18(10):966-73.

27. Severin F, Stollenwerk B, Holinski-Feder E, Meyer E, Heinemann V, Giessen-Jung C, et al. Economic evaluation of genetic screening for Lynch syndrome in Germany. Genet Med. 2015;17(10):765-73.

28. Snowsill T, Huxley N, Hoyle M, Jones-Hughes T, Coelho H, Cooper C, et al. A model-based assessment of the cost-utility of strategies to identify Lynch syndrome in early-onset colorectal cancer patients. BMC Cancer. 2015;15:313.

29. Pastorino R, Basile M, Tognetto A, Di Marco M, Grossi A, Lucci-Cordisco E, et al. Cost-effectiveness analysis of genetic diagnostic strategies for Lynch syndrome in Italy. PLoS One. 2020;15(7):e0235038.

30. Kang YJ, Killen J, Caruana M, Simms K, Taylor N, Frayling IM, et al. The predicted impact and cost-effectiveness of systematic testing of people with incident colorectal cancer for Lynch syndrome. Med J Aust. 2020;212(2):72-81.

31. Guzauskas GF, Jiang S, Garbett S, Zhou Z, Spencer SJ, Snyder SR, et al. Cost-effectiveness of population-wide genomic screening for Lynch syndrome in the United States. Genet Med. 2022;24(5):1017-26.

32. Snowsill TM, Ryan NAJ, Crosbie EJ. Cost-Effectiveness of the Manchester Approach to Identifying Lynch Syndrome in Women with Endometrial Cancer. J Clin Med. 2020;9(6).

33. Azardoost H, Rahimi F, Zeinalian M, Rezayatmand R. Cost-Effectiveness Analysis of Molecular Screening to Identify Lynch Syndrome in the Patients with Colorectal Cancer. Int J Cancer Manag. 2021;14(4).

34. Kunst N, Stout NK, O'Brien G, Christensen KD, McMahon PM, Wu AC, et al. Population-Based Newborn Screening for Germline TP53 Variants: Clinical Benefits, Cost-Effectiveness, and Value of Further Research. J Natl Cancer Inst. 2022;114(5):722-31.

35. Ngeow J, Liu C, Zhou K, Frick KD, Matchar DB, Eng C. Detecting Germline PTEN Mutations Among At-Risk Patients With Cancer: An Age- and Sex-Specific Cost-Effectiveness Analysis. J Clin Oncol. 2015;33(23):2537-44.

36. Yeh JM, Stout NK, Chaudhry A, Christensen KD, Gooch M, McMahon PM, et al. Universal newborn genetic screening for pediatric cancer predisposition syndromes: model-based insights. Genet Med. 2021;23(7):1366-71.

37. O'Brien G, Christensen KD, Sullivan HK, Stout NK, Diller L, Yeh JM, et al. Estimated Cost-effectiveness of Genetic Testing in Siblings of Newborns With Cancer Susceptibility Gene Variants. JAMA Netw Open. 2021;4(10):e2129742.

38. Ademi Z, Watts GF, Pang J, Sijbrands EJ, van Bockxmeer FM, O'Leary P, et al. Cascade screening based on genetic testing is cost-effective: evidence for the implementation of models of care for familial hypercholesterolemia. J Clin Lipidol. 2014;8(4):390-400.

39. Chen CX, Hay JW. Cost-effectiveness analysis of alternative screening and treatment strategies for heterozygous familial hypercholesterolemia in the United States. Int J Cardiol. 2015;181:417-24.

40. Crosland P, Maconachie R, Buckner S, McGuire H, Humphries SE, Qureshi N. Cost-utility analysis of searching electronic health records and cascade testing to identify and diagnose familial hypercholesterolaemia in England and Wales. Atherosclerosis. 2018;275:80-7.

41. Kerr M, Pears R, Miedzybrodzka Z, Haralambos K, Cather M, Watson M, et al. Cost effectiveness of cascade testing for familial hypercholesterolaemia, based on data from familial hypercholesterolaemia services in the UK. Eur Heart J. 2017;38(23):1832-9.

42. Lazaro P, Perez de Isla L, Watts GF, Alonso R, Norman R, Muniz O, et al. Cost-effectiveness of a cascade screening program for the early detection of familial hypercholesterolemia. J Clin Lipidol. 2017;11(1):260-71.

43. McKay AJ, Hogan H, Humphries SE, Marks D, Ray KK, Miners A. Universal screening at age 1-2 years as an adjunct to cascade testing for familial hypercholesterolaemia in the UK: A cost-utility analysis. Atherosclerosis. 2018;275:434-43.

44. Pelczarska A, Jakubczyk M, Jakubiak-Lasocka J, Banach M, Mysliwiec M, Gruchala M, et al. The cost-effectiveness of screening strategies for familial hypercholesterolaemia in Poland. Atherosclerosis. 2018;270:132-8.

45. Martin AC, Hooper AJ, Norman R, Nguyen LT, Burnett JR, Bell DA, et al. Pilot study of universal screening of children and child-parent cascade testing for familial hypercholesterolaemia in Australia. J Paediatr Child Health. 2022;58(2):281-7.

46. Catchpool M, Ramchand J, Martyn M, Hare DL, James PA, Trainer AH, et al. A cost-effectiveness model of genetic testing and periodical clinical screening for the evaluation of families with dilated cardiomyopathy. Genet Med. 2019;21(12):2815-22.

47. Brough J, Jain M, Jerves T, Kruszka P, McGuffey E. Genetic screening for hypertrophic cardiomyopathy in large, asymptomatic military cohorts. Am J Med Genet C Semin Med Genet. 2020;184(1):124-8.

48. Compagni A, Melegaro A, Tarricone R. Genetic screening for the predisposition to venous thromboembolism: a cost-utility analysis of clinical practice in the Italian health care system. Value Health. 2013;16(6):909-21.

49. Sutherland CS, Ademi Z, Michaud J, Schur N, Lingg M, Bhadhuri A, et al. Economic evaluation of a novel genetic screening test for risk of venous thromboembolism compared with standard of care in women considering combined hormonal contraception in Switzerland. BMJ Open. 2019;9(11):e031325.

50. de Graaff B, Neil A, Si L, Yee KC, Sanderson K, Gurrin L, et al. Cost-Effectiveness of Different Population Screening Strategies for Hereditary Haemochromatosis in Australia. Appl Health Econ Health Policy. 2017;15(4):521-34.

51. Richardson JS, Kemper AR, Grosse SD, Lam WKK, Rose AM, Ahmad A, et al. Health and economic outcomes of newborn screening for infantile-onset Pompe disease. Genet Med. 2021;23(4):758-66.

52. Bennette CS, Gallego CJ, Burke W, Jarvik GP, Veenstra DL. The cost-effectiveness of returning incidental findings from next-generation genomic sequencing. Genet Med. 2015;17(7):587-95.

53. Zhang L, Bao Y, Riaz M, Tiller J, Liew D, Zhuang X, et al. Population genomic screening of all young adults in a health-care system: a cost-effectiveness analysis. Genet Med. 2019;21(9):1958-68.

54. Jittikoon J, Sangroongruangsri S, Thavorncharoensap M, Chitpim N, Chaikledkaew U. Economic impact of medical genetic testing on clinical applications in Thailand. PLoS One. 2020;15(12):e0243934.
